# Apelin increases atrial conduction velocity, refractoriness, and prevents inducibility of atrial fibrillation

**DOI:** 10.1172/jci.insight.126525

**Published:** 2020-09-03

**Authors:** Young M. Kim, Robert Lakin, Hao Zhang, Jack Liu, Ayaaz Sachedina, Maneesh Singh, Emily Wilson, Marco Perez, Subodh Verma, Thomas Quertermous, Jeffrey Olgin, Peter H. Backx, Euan A. Ashley

**Affiliations:** 1 Division of Cardiovascular Medicine, Stanford Medicine, Stanford, California, USA.; 2 Department of Biology, York University, Toronto, Ontario, Canada.; 3 Division of Cardiology, University Health Network, Toronto, Ontario, Canada.; 4 Division of Cardiovascular Medicine, University of California, San Francisco, San Francisco, California, USA.

**Keywords:** Cardiology, Arrhythmias

## Abstract

Previous studies have shown an association between elevated atrial NADPH-dependent oxidative stress and decreased plasma apelin in patients with atrial fibrillation (AF), though the basis for this relationship is unclear. In the current study, RT-PCR and immunofluorescence studies of human right atrial appendages (RAAs) showed expression of the apelin receptor, *APJ*, and reduced apelin content in the atria, but not in plasma, of patients with AF versus normal sinus rhythm. Disruption of the apelin gene in mice increased (2.4-fold) NADPH-stimulated superoxide levels and slowed atrial conduction velocities in optical mapping of a Langendorff-perfused isolated heart model, suggesting that apelin levels may influence AF vulnerability. Indeed, in mice with increased AF vulnerability (induced by chronic intense exercise), apelin administration reduced the incidence and duration of induced atrial arrhythmias in association with prolonged atrial refractory periods. Moreover, apelin decreased AF induction in isolated atria from exercised mice while accelerating conduction velocity and increasing action potential durations. At the cellular level, these changes were associated with increased atrial cardiomyocyte sodium currents. These findings support the conclusion that reduced atrial apelin is maladaptive in fibrillating human atrial myocardium and that increasing apelin bioavailability may be a worthwhile therapeutic strategy for treating and preventing AF.

## Abstract 2

Reduced apelin is maladaptive in fibrillating human atrial myocardium, and increasing apelin bioavailability may be a potential therapeutic strategy for treating and preventing AF.

## Introduction

Atrial fibrillation (AF) is the most common clinical arrhythmia and is the leading cause of disabling stroke (1). As the prevalence of AF is increasing due to an aging population (2), AF exerts an increasing strain on the health care system (2, 3). Current management of AF focuses on both medical and catheter ablation–directed strategies (4). However, as a dynamic disease process, AF clinically presents as a continuum from paroxysmal to permanent AF, with complex molecular, ionic, and cellular structural remodeling as the disease progresses. Electrophysiological remodeling in AF includes shortened atrial refractoriness and slowed conduction, which may be related to elevated atrial oxidative stress (5–7), and strategies to increase conduction velocity (CV) and/or increase refractoriness may protect against AF initiation, maintenance, and recurrence (8–10).

Apelin is an endogenous peptide hormone with pyroglutamylated apelin-13 (^PYR^apelin^13^) being the predominant circulating form (11, 12) that signals via the G protein–coupled receptor, *APJ* (APLNR). Although apelin plays a pivotal role in cardiac development and angiogenesis (13), it also strongly directly and indirectly influences cardiovascular physiology. For example, apelin antagonizes the renin-angiotensin-aldosterone system (14–18), thereby altering blood pressure (18, 19) and cardiac inotropy (20, 21) as well as myocardial fibrosis, hypertrophy, and oxidative stress (14, 15). On the other hand, *APJ* receptors have been shown to directly regulate ventricular excitability, conduction, contractility, and refractoriness (22, 23) in association with increased cardiac peak sodium current and slowed recovery from inactivation (23). Interestingly, apelin immunoreactivity is 200-fold greater in atrial versus ventricular tissue (23, 24), suggesting a role in the regulation of atrial electrophysiology. Moreover, plasma apelin levels are reduced in patients with lone AF (25) as well as other supraventricular arrhythmias (26), and plasma apelin levels predict atrial arrhythmia recurrence (27), suggesting that apelin might have a benefit in treating atrial arrhythmias. In this regard, we previously demonstrated that patients with AF have elevated atrial NADPH oxidase activity and oxidative stress (6), which has been associated decreased apelin levels (14). Therefore, we set out to directly examine connections between apelin and atrial electrophysiological properties as well as arrhythmias.

## Results

### Apelin and apelin receptors in human atria.

**Figure 1:**
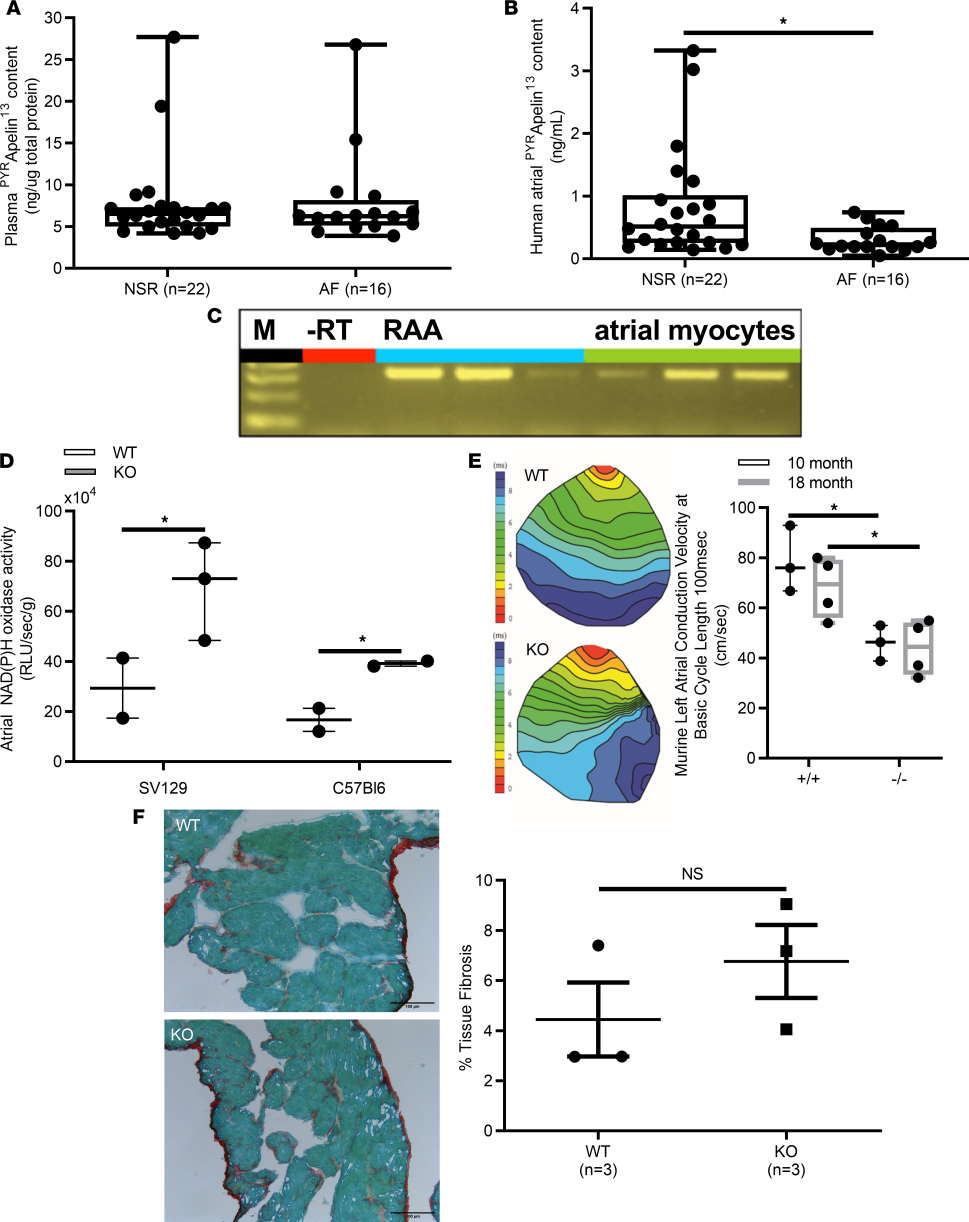
Molecular characterization of human apelin receptor, APJ, in human right atrial appendage and relationship between atrial and plasma apelin content with atrial NADPH oxidase activity. (**A**) Plasma ^PYR^apelin^13^ content was not statistically different in patients with normal sinus rhythm (NSR) versus atrial fibrillation (AF) (7.85 ± 1.2 ng/μg total protein vs. 8.12 ± 1.5 ng/μg total protein, *P* = 0.89). (**B**) By contrast, human atrial ^PYR^apelin^13^ content was significantly reduced in AF versus NSR (0.29 ± 0.05 ng/mL vs. 0.73 ± 0.2 ng/mL, *P < 0.05). (**C**) Reverse transcriptase PCR (RT-PCR) for human *AJP* in both human right atrial appendage (RAA) (blue lanes) and isolated atrial myocytes (green lanes) from patients with NSR yielded the calculated 323 bp fragment in 3 patients. M represents the 100 kb ladder. (**D**) In 2 independent murine strains, 129SV and C57BL6 homozygous deletion of the apelin gene versus WT controls resulted in intact atrial NADPH oxidase activity, which was approximately 2.4-fold greater (*n* = 3 animals per group, *P < 0.05). (**E**) Representative atrial CV isochrones derived from pacing of the left atrial myocardium at pacing cycling lengths from 250 to 80 ms in apelin WT and -KO mice. Ex vivo murine left atrial voltage-sensitive optical mapping microscopy with di-4-ANEPPS revealed a significant decrease in atrial CV in apelin-KO versus WT control mice (*n* = 4 at 18 months of age, *n* = 3 at 10 months of age, *P < 0.05 versus WT, +/+). (**F**) Representative Sirius red staining demonstrating no significant changes in atrial interstitial fibrosis between WT (4.44% ± 1.48%) and apelin-KO mice (6.76% ± 1.46%) (scale bar: 100 mm). RLU, relative light units. Student’s *t* test, *P < 0.05.

Given that plasma apelin levels have been shown to be decreased in patients with lone (25) and persistent AF (27), we began by measuring plasma apelin levels in patients. As shown in Figure 1A, plasma apelin levels were similar (*P* = 0.71) in patients with AF (8.0 ± 1.4 pg/mL, *n* = 16) and patients with normal sinus rhythm (NSR) (7.8 ± 1.2 pg/mL, *n* = 22). However, atrial apelin levels were reduced (*P* < 0.05) approximately 2.3-fold in human atrial myocardium from patients with AF (0.3 ± 0.1 ng/mL) compared with those in patients with NSR (0.8 ± 0.2 ng/mL) (Figure 1B). To assess the potential relevance of reduced atrial apelin levels, we looked for evidence of apelin receptor (i.e., APJ receptor) expression in the right atrial myocardium of patients undergoing cardiac surgery (see Table 1 for patient characteristics). Figure 1C confirms the presence of apelin receptor mRNA in right atrial homogenates (11), while Supplemental Figure 1 (supplemental material available online with this article; https://doi.org/10.1172/jci.insight.126525DS1; see complete unedited blots in the supplemental material) establishes punctate expression of the APJ receptor in isolated human atrial myocytes, along with some diffuse staining. Connexin-43 staining was also performed to determine the architectural integrity of our isolated human atrial cardiomyocytes, and these results confirmed the expected localization pattern. Moreover, omission of the primary antibody (negative control) resulted in no relevant staining in 9 of 9 cells from 3 patients.

### Effects of apelin gene disruption in mice.

Since we previously established that AF is associated with elevated oxidative stress as a result of increased NADPH oxidase activity (6) and apelin has been shown to negatively regulate oxidative stress (28), we measured atrial NADPH oxidase activity in WT and apelin-KO mice. In line with the human results, NADPH oxidase activity was increased (*P* < 0.05) in apelin-KO mice compared with WT controls when measured in mice with the C57BL6 or SV129 genetic background (Figure 1D). Moreover, C57BL6 apelin-KO mice showed reduced atrial CVs compared with WT controls at both 10 months (*P* < 0.05, *n* = 3) and 18 months (*P* < 0.05, *n* = 3) (Figure 1E). Despite these differences in CV, Sirius red staining revealed no differences (*P* = 0.327) in collagen levels between WT (4.44% ± 1.48%) and apelin-KO mice (6.76% ± 1.46%), suggesting that the reduced CV might arise from electrical changes in the atrial myocardium (Figure 1F).

### Apelin increases atrial CV and refractoriness and protects against atrial arrhythmia inducibility.

**Figure 2:**
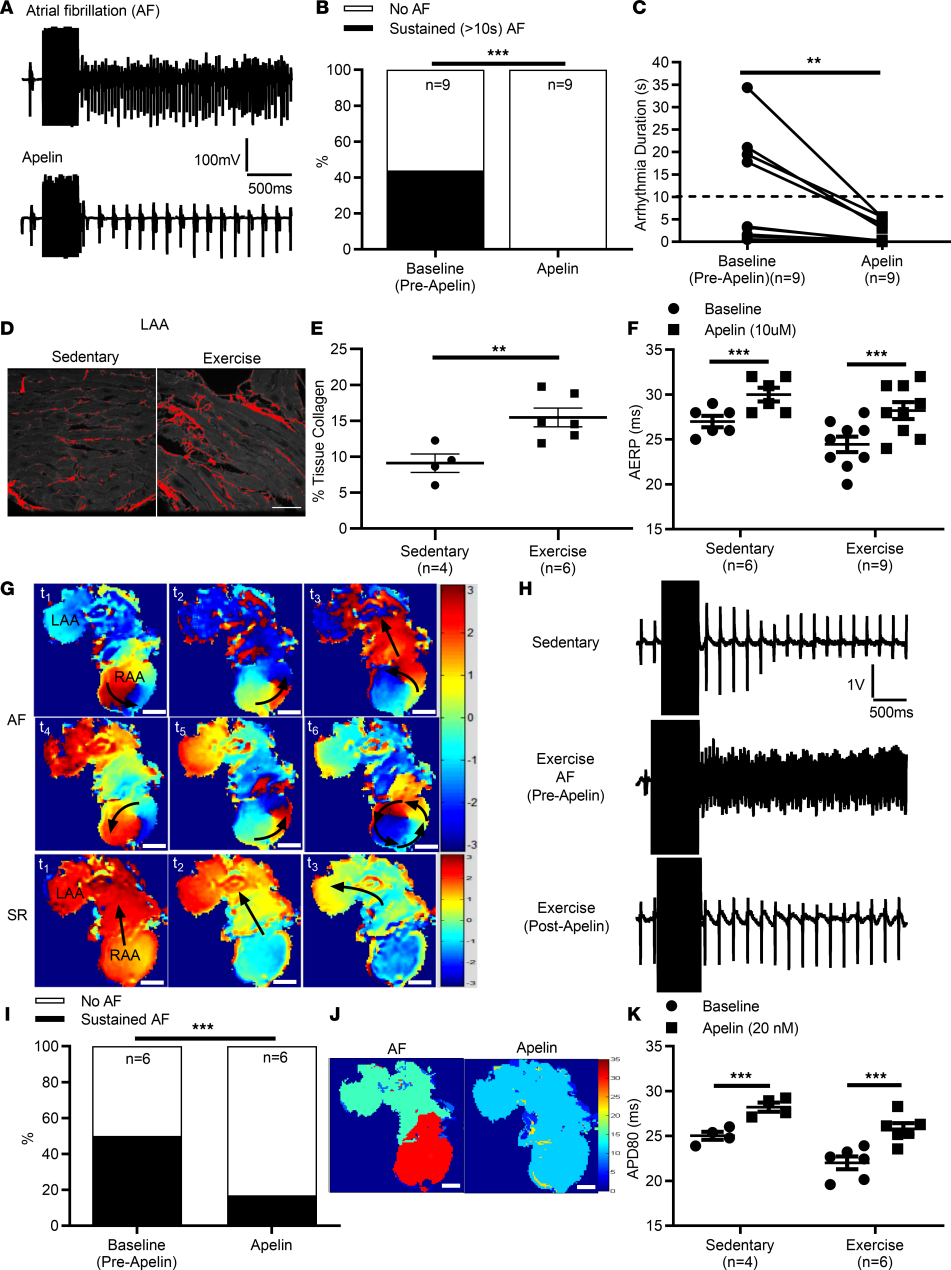
Direct in vivo and ex vivo effects of ^PYR^apelin^13^ on atrial arrhythmia inducibility on swim-trained mice. Vulnerability to atrial arrhythmias was determined in vivo using an intracardiac octapolar catheter placed in the right ventricle, followed by programmed electrical stimulations. (**A**) Representative intracardiac bipolar electrograms showing inducibility of atrial fibrillation (AF) before and following ^PYR^apelin^13^ (10 μM i.p.) injection in exercised mice. (**B**) The percentage of exercised mice with sustained (>10 s) AF (44% vs. 0%, *P* < 0.0001) as well as arrhythmia durations (*P* = 0.0039) decreased (**C**) following ^PYR^apelin^13^ administration. (**D** and **E**) Intense exercise caused elevated (*P* = 0.0103) atrial fibrosis compared with sedentary mice. (**F**) Decreased arrhythmia vulnerability with ^PYR^apelin^13^ was associated with prolongation (~16%, *P* = 0.0004) of the atrial effective refractory period (AERP), which was also observed in sedentary control mice (~11%). (**G**) Vulnerability to arrhythmias was determined ex vivo in isolated (denervated) atrial preparations from swim-trained (*n* = 6) and sedentary control (*n* = 4) mice. Representative field recordings showing the baseline and inducibility of atrial arrhythmias before and following ^PYR^apelin^13^ (20 nM) administration. (**H**) A typical representative AF event initiated in the right atrial appendage (RAA) by programmed electrical stimulation recorded by optical mapping of atria loaded with Di-4-ANEPPS showing rotor formation in the RAA, characterized by rapid focal activity, reentry circuits, and 2:1 conduction block. AF inducibility was decreased (*P* < 0.0001) following ^PYR^apelin^13^ administration, with normal conduction observed with 90 ms pacing at the RAA. (**I**) The percentage of exercised mice with sustained (>10 s) atrial arrhythmias decreased (50% to 17%, *P* < 0.0001) following ^PYR^apelin^13^ administration, consistent with a decrease (*P* = 0.048) in arrhythmia durations (13.1 ± 4.1 s vs. 3.3 ± 1.7 s). (**J**) Representative dominant frequency (DF) maps (Hz) generated from the time dependence of each pixel intensity subjected to a moving fast Fourier transform analysis. During AF, the local DF of electrical activity was observed within the RAA. Following ^PYR^apelin^13^ administration, the DF in the RAA was eliminated, consistent with an inability to induce atrial arrhythmias. (**K**) Decreased arrhythmia inducibility following ^PYR^apelin^13^ administration was associated with action potential duration prolongation (~17%, *P* < 0.001) at 80% of repolarization (APD80). APD80 also increased (~13%, *P* < 0.001) in sedentary mice, in which atrial arrhythmias cannot be induced. Data are presented as mean ± SEM. Scale bar: 200 μm. Representative data are the result of experiments being repeated at least 6 times. *P < 0.05, **P < 0.01, ***P < 0.001, paired and unpaired 2-tailed Student’s *t* tests, χ^2^ test, Mann-Whitney *U* or Wilcoxon signed-rank test comparing before and after ^PYR^apelin^13^ administration.

To examine the effects of apelin on atrial electrophysiology, apelin (10 μM, i.p.) was administered to intensely exercised mice, which we have shown have increased vulnerability to atrial arrhythmia (fibrillation and flutter) induction (29, 30). As expected, swim-exercised mice (*n* = 9) showed increased atrial arrhythmias. More importantly, apelin decreased (*P* < 0.0001) the incidence arrhythmia inducibility (~44% to 0%) in association with decreased (*P* = 0.0039) arrhythmia duration (11.4 ± 4.0 s vs. 2.1 ± 0.8 s) compared with baseline (before apelin) (Figure 2, A–C). Indeed, we found that increased AF vulnerability in swim-exercised mice was associated with elevated (*P* = 0.0103) atrial fibrosis (Figure 2, D and E), as reported previously (29). Consistent with its effects on AF inducibility, apelin administration resulted in prolongation (*P* = 0.0004) of the atrial effective refractory period by approximately 16% (Figure 2F) in the atria of exercised mice, an effect that was also observed in control sedentary mice (~11% increase, *P* < 0.0001, *n* = 6).

**Figure 3:**
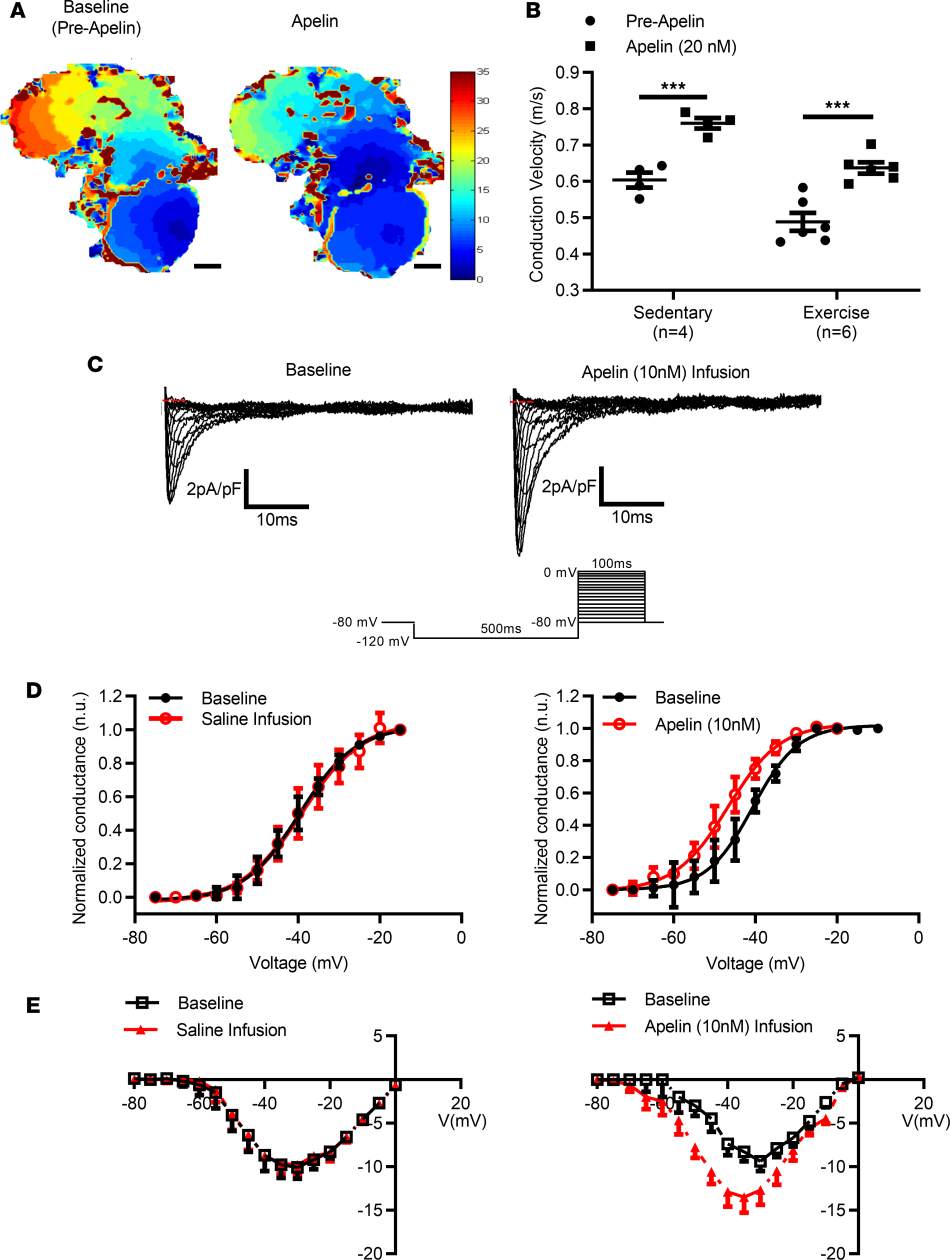
Direct functional effects of ^PYR^apelin^13^ on atrial electrophysiology. (**A**) Representative murine atrial activation isochrones (ms) at baseline and following ^PYR^apelin^13^ administration (20 nM) in isolated (denervated) atria paced at 90 ms intervals from the right atrial appendage (RAA) (scale bar: 200 μm). (**B**) Conduction velocities calculated from the left atrial appendage (LAA) of 90 ms paced atria were increased (***P < 0.0007, 2-tailed Student’s paired *t* test comparing before and after ^PYR^apelin^13^ administration) following ^PYR^apelin^13^ administration in both exercised and sedentary control mice. (**C**) Representative tracings and I–V relationships of INa from isolated murine left atrial (LA) myocytes before (baseline) and after ^PYR^apelin^13^ (10 nM) infusion. (**D**) Whole-cell voltage-clamp steady-state sodium channel activation curves were determined from single-cell recordings from sedentary CD1 mice (8–10 weeks old) of cardiac sodium current (I_Na_) in freshly isolated murine LA myocytes before and after treatment with ^PYR^apelin^13^ (10 nM) infusion and saline (control). In contrast to saline infusion, apelin reversibly induced a hyperpolarized voltage shift (*P* = 0.041) in the steady-state activation curve from –40.68 ± 2.03 mV to –46.58 ± 1.69 mV. (**E**) I–V relationship of I_Na_ from LA myocytes before and after saline revealed no change in maximal conductance. By contrast, ^PYR^apelin^13^ (10 nM) infusion increased (*P* = 0.006) maximum conductance from 0.34 ± 0.02 pS/pF to 0.41 ± 0.02 pS/pF. Data are presented as mean ± SEM. For sodium channel measures, *n* = 7 myocytes/3 mouse isolated atria per group. Representative data are the result of experiments being repeated 6 times.

To assess whether these effects of apelin resulted from direct actions on the atrial myocardium, we also exposed isolated (denervated) atria to apelin (20 nM). As expected, AF was readily inducible (3 of 6, 50%) in atria from exercised mice and was characterized by dynamic and complex features, involving rapid focal activity, wavelets, reentry rotors, and conduction block (Figure 2, G and H). Apelin reduced (*P* < 0.0001) arrhythmia inducibility (Figure 2I) and decreased (*P* = 0.048) arrhythmia durations (13.1 ± 4.1 s vs. 3.3 ± 1.7 s) in atria isolated from exercised mice. Moreover, when arrhythmias were observed in the presence of apelin, the dominant frequencies were reduced (Figure 2J); as expected, none of the sedentary control mice showed evidence of inducibility (data not shown). The ability of apelin to reduce arrhythmia vulnerability in isolated exercised atria was associated with prolongation (~17%, *P* < 0.001) of action potential duration at 80% of repolarization (APD80) (Figure 2K), which was also observed in atria isolated from sedentary control mice (in which atrial arrhythmias cannot be induced) (~13%, *P* < 0.001). Consistent with studies in apelin-KO mice, apelin (20 nM) also increased (*P* < 0.0007) CVs in atria isolated from both exercised and sedentary control mice (Figure 3, A and B) as well as in cultured monolayer of murine HL-1 atrial myocytes from 1.6 ± 0.02 cm/s to 2.2 ± 0.02 cm/s (*P* = 0.006) (Supplemental Figure 2), an effect which was reversed on washout.

Since the effects of apelin appeared to involve a cell-autonomous mechanism, we considered that the effects of apelin on CV (and possibly AP prolongation) were mediated by changes in atrial cardiomyocyte sodium currents. Whole-cell voltage-clamp recordings from freshly isolated murine left atrial myocytes from sedentary CD1 mice (8–10 weeks old) revealed that, in contrast to saline infusion, apelin reversibly induced a hyperpolarized voltage shift (*P* = 0.041) in the steady-state activation curve from –40.68 ± 2.03 mV to –46.58 ± 1.69 mV, in conjunction with increases (*P* = 0.006) in the maximum conductance from 0.34 ± 0.02 pS/pF to 0.41 ± 0.02 pS/pF (*n* = 7 myocytes averaged per 3 mouse isolated atria per group) (as summarized in Figure 3, C–E). No differences in steady-state inactivation were observed following apelin administration (data not shown).

## Discussion

The main findings of this study are as follows: (a) apelin content is reduced in the atria, but not the plasma, of patients with AF; (b) human atrial myocytes express the molecular APJ receptor in a largely punctate pattern; (c) atrial NADPH-dependent superoxide production is increased in apelin-KO mice; (d) apelin increases CV both in atrial myocyte monolayers and in the intact murine left atrium; (e) apelin augments sodium current in isolated atrial cardiomyocytes; (f) apelin increases the atrial refractory period in vivo by approximately 16% and action potential durations (APDs) by approximately 17% ex vivo; and (g) apelin protects against atrial arrhythmia vulnerability in intensely exercised mice. Taken together, our findings establish that apelin can modify atrial electrophysiology and reduce AF inducibility, thereby suggesting that reversing the reduced apelin levels in patients with AF may be an effective therapeutic strategy for treating AF.

### Apelin and APJ in human atrial myocardium.

In the current study, we provide the first evidence to our knowledge of the presence of human atrial apelin receptor, *APJ*, by RT-PCR and immunofluorescence in isolated human right atrial myocytes and tissue homogenates of RAAs of patients undergoing cardiac surgery. In particular, we observed a punctate staining of apelin receptor, *APJ*, expression in human atrial cardiomyocytes, consistent with the presence of APJ in atrial myocytes as well as the results of previous studies, which found localization of the APJ receptor at the Z-lines (22, 23, 31, 32) of ventricular cells.

As observed previously in ventricles of patients with heart failure (33), we found reduced apelin levels in right atrial homogenates from patients with AF, suggesting that decreased apelin-APJ signaling may contribute to the maintenance and pathophysiology of AF. On the other hand, these reductions in atrial apelin levels were not associated with reduced plasma apelin in patients with AF, despite previous studies reporting reductions in plasma apelin of patients with lone (25) and persistent (34) AF with as well as patients with heart failure (33). The lack of differences in apelin between patients with AF and NSR in our study compared with those in other studies may reflect differences in overall disease progression, which might suggest that atrial apelin levels precede reductions in plasma levels depending on the underlying AF pathogenesis.

We have previously documented that patients with paroxysmal or persistent AF have increased atrial NADPH oxidase activity and that atrial NADPH oxidase activity predicts the occurrence of AF following cardiac surgery (7). Since the apelin-APJ system regulates electrophysiology (22), oxidative stress (28), and adverse cardiac remodeling (14), we sought to determine whether apelin may regulate atrial oxidative stress and promote AF. Our findings indicate that atrial NADPH oxidase activity in apelin-KO mice was significantly increased compared with that in WT controls in mice with either the C57BL6 or SV129 genetic background. These findings are consistent with those of previous studies showing that apelin decreases superoxide production in cardiomyocytes (28), possibly decreasing renin-angiotensin-aldosterone system activity (14), which is predicted to reduce hypertrophic and fibrotic cardiac remodeling, both of which promote AF (35). As enhanced atrial superoxide production is a self-perpetuating process (36) that increases myocardial oxygen consumption, β-adrenergic responsiveness, and thrombogenic risk linked to AF (37–39), our findings suggest that increasing atrial apelin bioavailability may represent a strategy for preventing and treating AF. However, while we observed enhanced atrial oxidative stress, we did not observe atrial interstitial fibrosis in apelin-KO mice, suggesting that other reduced apelin alone is insufficient to promote AF.

### Direct effects of apelin on electrophysiology, arrhythmogenic ionic and structural substrate, and therapeutic protective effect against AF inducibility.

Our results confirm that apelin administration has direct effects on atrial electrophysiology that are predicted to reduce AF vulnerability. Specifically, we observed reductions in CV in atria from apelin-KO mice in the absence of atrial interstitial fibrosis. Augmentation of CV was also observed following acute apelin application to (denervated) atria isolated from sedentary and swim-exercised mice as well as to cultured monolayers of murine Hl-1 atrial myocytes. The ability of apelin to augment CV and excitability is consistent with findings of previous studies (22, 23). Additionally, apelin increased both in vivo atrial effective refractory periods and prolonged atrial APDs in sedentary and swim-trained mice, in agreement with the effects of apelin on ventricular myocytes (23). Given these findings, we further tested whether apelin treatment could limit or prevent AF in our intense swim exercise model of AF (29, 30). We previously showed that intense exercise of mice results in increased atrial arrhythmia inducibility in intact mice as well as in isolated atria, characterized by rapid focal activity, wavelets, reentry rotors, and conduction block (29). Consistent with its electrical actions, apelin treatment decreased atria arrhythmia inducibility and duration in vivo from exercised mice. Apelin was also able to reduce AF inducibility and duration in atria isolated from exercised mice, and this was associated reduced dominant frequencies in optical mapping studies, as would be expected with increased CV and prolonged APD. Our results show a clear link between apelin bioavailability and atrial CV in apelin-KO mice and rescue with acute apelin administration. However, whether acute apelin administration reverses reductions in CV and prolongs APDs in apelin-KO mice warrants further study.

Since the effects of apelin appeared to involve a cell-autonomous mechanism, we considered that the effects of apelin on CV were mediated by changes in atrial cardiomyocyte sodium currents (I_Na_). Consistent with findings of previous studies in both atrial (24) and ventricular myocytes (21, 23), we found that exposing left atrial myocytes to apelin increased the maximal I_Na_ conduction in association with a reversible hyperpolarizing shift in steady-state sodium channel activation combined with AP prolongation. This latter finding can help explain the ability of apelin to increase atrial refractoriness and prolong APDs, as reported previously (40, 41)), actions which are expected to reduce arrhythmia vulnerability (42).

While our results aligns with the effects of apelin on I_Na_ and APDs in ventricular myocytes (23), they differ from a previous report (24) concluding that apelin reduces APDs in atrial myocytes in association with increased I_Kur_ and decreased I_Ca,L_, despite increasing I_Na_. On the other hand, other studies have concluded that apelin has no effects on L-type calcium currents or voltage-gated potassium currents (21, 43). These findings combined with our own suggest that elevations in I_Na_ might underlie APD prolongation in our studies, presumably as a result of increased late sodium current (44). Nonetheless, we cannot rule out the potential contribution of changes in other currents to the AP changes induced by apelin application. Future studies will be needed to determine the basis the effects of apelin on APD prolongation as well as on AF inducibility.

### Limitations.

The findings of our study should be interpreted in the context of their limitations. The assessment of human atrial apelin content was performed on samples from the RAA, and apelin content may be heterogeneous across the atrial myocardium due to tissue unavailability. As the apelin receptor is similar to the angiotensin I and II receptors, which are regionalized in the human atrial myocardium, the apelin receptor may also prove to have regional differences in the human atria and, therefore, consequently exert anatomically site-specific roles; thus, caution should be exerted in extrapolating our findings to the whole atrial myocardium. Given the paucity of human NSR and AF specimens in the current study, future studies will be needed to further quantify APJ expression and its localization as well as to assess alterations in conditions such as AF. Moreover, while our findings suggest an association between apelin bioavailability and oxidative stress, the connection between this association and the pathogenesis of AF will also need to be investigated further in NSR and AF atrial tissues as well as possibly in apelin-KO mice. Finally, while our results suggest that apelin alters APD (and the effective refractory period) through its effects on I_Na_, we cannot rule out the contribution of other ion channels to apelin-mediated AP changes.

### Clinical implications.

To our knowledge, our study provides the first evidence of insufficient apelin bioavailability in the human atrial myocardium in AF. In addition, our study is possibly the first to show that apelin directly modulates arrhythmia inducibility, with acute administration protecting against reentry arrhythmias in vivo and ex vivo by increasing both CV and APD. Taken together, these findings indicate for the first time to our knowledge that reduced atrial-specific apelin bioavailability in the fibrillating atrial myocardium contributes to enhanced atrial oxidative stress, removes an endogenous mechanism to maintain normal atrial CV, and, thus, may play an important role in the vulnerability and treatment of atrial oxidative injury and electrophysiological remodeling observed in patients with AF. Our findings suggest that reduced apelin bioavailability may represent a maladaptive phenomenon in AF and that increasing apelin bioavailability may be a potential therapeutic target to overcome atrial oxidative injury and electrophysiological remodeling observed in patients with AF.

## Methods

Further details on the methods below can be found in the Supplemental Methods.

### Patient characteristics.

Experiments designed to document the presence of the APJ receptor were performed in tissue samples from 9 patients with NSR for immunolocalization and RT-PCR studies. In addition, we evaluated atrial ^PYR^apelin^13^ content in RAA homogenates and plasma obtained from 16 patients with a history of AF and 22 patients with NSR with no previous history of arrhythmias, who were matched for treatment, age, and other known risk factors for cardiovascular disease. The AF group included 7 patients who had permanent AF and 9 who had a history of symptomatic paroxysmal AF or persistent AF. We grouped these patients together because of evidence indicating that paroxysmal AF in humans is associated with electrophysiological and structural changes of the atrial myocardium that are similar to those observed in patients with permanent AF (45–47), suggesting that intermittent bursts of AF, particularly if frequent, are sufficient to instigate atrial remodeling in humans. Demographic and clinical characteristics of the patient groups are shown in Table 1.

### Human atrial myocyte isolation protocol.

Samples of RAA were digested (40 minutes) in isolation buffer supplemented with type I collagenase (2 mg/mL; Worthington) and protease (0.2 mg/mL; MilliporeSigma), 1% BSA, and 50 μmol/L Ca^2+^ and then transferred into fresh collagenase solution without protease. After isolation, rod-shaped atrial myocytes were enriched (to ≈90%) by a 2-step centrifugation (3 minutes at ≈18*g*) in Kraft-Bruhe medium supplemented with L-glutamate, sodium pyruvate, taurine, creatine, succinic acid, Na_2_ATP, and β-OH butyrate and used within 3 hours for superoxide measurements or immunofluorescence confocal microscopy.

### Immunolocalization.

We established the molecular presence of the human atrial apelin-receptor, APJ, by RT-PCR and confocal immunofluorescence in isolated human right atrial myocytes and tissue homogenates of RAAs from patients undergoing cardiac surgery in normal sinus rhythm (*n* = 22) and AF (*n* = 16). Freshly isolated human atrial myocytes were concentrated by centrifugation (≈18*g*; 3 minutes). Myocytes were plated on laminin-coated (60 μg/mL) coverslips and fixed (1.5 hours at room temperature) by applying 50 μL cooled 4% paraformaldehyde containing 0.2% Triton X-100. Coverslips were washed with PBS 3 times and then blocked (2 hours at room temperature) with 5% BSA. Concentrations were optimized and cells were incubated with the primary antibody (1 hour at room temperature; rabbit polyclonal APJ) diluted 1:500 in PBS containing 5% BSA, washed in PBS, and then incubated (30 minutes) with 1:50 fluorescence-conjugated goat anti-rabbit IgG (Alexis). Fluorescence images were obtained with a Leica scanning confocal microscope.

### RT-PCR.

RT-PCR was used to evaluate the presence of human APJ receptor in total RNA extracted from RAA homogenates and isolated atrial myocytes from patients with NSR using Trizol reagent (Gibco). The PCR primers were 5′CACCTGGTGAAGACGCTGA3′ and 5′TAGGGGATGGATTTCTCGTG3′, yielding a 323 bp fragment (48). Reverse transcription was performed on 1 μg total RNA (Promega). Optimal PCR conditions were as follows: 94°C for 1 minute, 58°C for 45 seconds, and 72°C for 1 minute for 35 cycles followed by a 10-minute incubation at 72°C. The RT-PCR was resolved on 1.5% agarose ethidium bromide containing gels. The identities of PCR products were confirmed by direct sequencing using an automatic DNA sequencer (PE Applied Biosystems).

### Atrial NADPH oxidase activity and apelin measurements.

Murine left atrial NADPH oxidase activity was measured by DPI and tiron-inhibitable lucigenin-enhanced chemiluminescence using a single-tube luminometer (Berthold FB12) modified to maintain the sample temperature at 37°C (6). Briefly, basal chemiluminescence from intact whole left atrium was measured in buffer (2 mL) containing lucigenin (5 μmol/L) after reaching equilibrium (≈7 minutes). Measurement of ^PYR^apelin^13^ content in human atrial myocardium and plasma were performed in a blinded fashion using a commercially available Phoenix Pharmaceuticals ELISA kit (EK05723) according to the manufacturer’s instructions.

### Swim exercise protocol.

Male CD1 mice (6 weeks old, Charles River) were randomized to swim (*n* = 9) and sedentary (*n* = 6) experimental groups. Swim training was performed, as outlined previously (30). Briefly, mice swam in containers (30 cm diameter) filled with water at 33°C circulating at 10–20 L/min. Swim training started with sessions lasting 30 minutes, which subsequently increased by 10 minutes per day until the session length reached 90 minutes. Thereafter, the mice swam for 90 minutes twice per day (separated by 4 hours), 5 days per week, for the following 6 weeks. Sedentary mice were placed in swim containers without water current twice per day to ensure similar handling.

### In vivo assessment of murine atrial arrhythmia vulnerability.

Male CD1 mice (*n* = 9) (6 weeks old, Charles River) that had undergone an intense 6-week swim exercise protocol, shown to drive adverse atrial structural remodeling and increased atrial arrhythmia inducibility (29) and their sedentary controls (*n* = 6), were used to assess the in vivo effects of ^PYR^apelin^13^ on arrhythmia vulnerability. Briefly, mice were anesthetized using 1.5% isoflurane and oxygen mixture and a 2.0 F octapolar catheter recording/stimulation electrophysiological catheter (CI’BER Mouse, Numed) inserted into the right jugular vein and advanced into the right ventricle. The bundle signal was used to confirm position of the catheter. Appropriate leads were used to deliver programmed stimulations to the right atria. All programmed electrical stimulations were delivered at a voltage of 1.5 times capture threshold, with a 1 ms pulse duration. Atrial effective refractory periods were determined by delivering 7 pulses at 20 ms below the R-R interval followed by an extrastimulation, with the S2 coupling interval initially delivered below capture (~15 ms) and increased until capture. For arrhythmia induction, 27 pulses (2 ms) at 40 ms intervals were applied to the right atrium and reduced by 20 ms intervals by 2 ms decrements, repeated 3 times. If arrhythmia events were not induced, 20 trains (applied at 1.5 s intervals) of 20 pulses (2 ms), with an interpulse interval of 20 ms duration, were used. Sustained arrhythmias were defined as reproducible episodes of rapid, chaotic, and continuous atrial activity, lasting longer than 10 seconds. Assessments were made before and following ^PYR^apelin^13^ (10 μM, i.p.) administration, with measurements made at 3 minutes following apelin administration given its short-half-life (<5 minutes) (49).

### Murine ex vivo left atrial optical mapping.

Optical mapping using the voltage-sensitive dye di-4-ANEPPS was performed in a blinded fashion on 7- to 8-week-old male SV129 and C57BL6 apelin-KO animals (Charles River), which we previously generated as described elsewhere (50), and littermate controls. Pacing was performed on an area of viable myocardium on the LA epicardium at pacing cycle lengths (PCLs) from 250 to 80 ms and then with S1-S2 using a basic PCL of 150 ms, with S2 decremented by 2 ms until the atrial effective refractory period was reached. Data analysis was performed by using custom algorithms MATLAB (MathWorks) programs. Isochronal activation maps and CV were analyzed as described previously (51, 52). Briefly, CV (velocity vectors) in the LA epicardium was determined by measuring 3–5 mapping sites along the direction of the propagating wavefront. The protocol consisted of a baseline recording, followed by infusion of ^PYR^apelin^13^ (20 nM) and washout.

For swim-trained (*n* = 6) and sedentary (*n* = 4) CD1 mice (Charles River), a different protocol was used, consistent with our previous work (29). Briefly, isolated atrial preparations were stained with 10 μM voltage-sensitive dye di-4-ANEPPS (MilliporeSigma) for 10 minutes and then superfused continuously with carbogenized Krebs solution (35°C). The flow rate, volume, and temperature of solution were kept constant throughout and in-between the experiments. The pH of the bath solution in the perfusion vessels was monitored to ensure a consistent pH (7.35–7.4). To calculate CV, atria were paced at a 90 ms interval from the RAA, and images were captured at 1000 frames/s using a high speed camera (MiCAM Ultima-L, SciMedia). Activation maps were generated to calculate CV in the left atrial appendage, with CV (velocity vectors) measured at 3–5 mapping sites along the direction of the propagating wavefront. APD was determined at 80% of repolarization (APD80). To induce atrial arrhythmias, the same stimulation protocols that were used in vivo (see above) were applied. Sustained arrhythmias were defined as a reproducible episode of rapid, chaotic atrial activation lasting longer than 10 seconds. Images were analyzed using BV_Analyze (Brainvision) and Rhythm software. All measurements were conducted before and 15 minutes after ^PYR^apelin^13^ superfusion (20 nM). Representative dominant frequency maps (Hz) generated from the time dependence of each pixel intensity subjected to a moving fast Fourier transform analysis. During AF, the local dominant frequency of electrical activity was observed within the RAA.

### Murine electrophysiological studies.

Left atrial myocytes for electrophysiology studies were isolated from sedentary CD1 mice (8–10 weeks old) following a previously published protocol (53) with modification. Briefly, hearts were rapidly removed from anesthetized (isoflurane [4%] oxygen mixture inhalation) mice and retrogradely perfused through the aorta for 3–4 minutes with 37°C Ca^2+^-free Tyrode’s solution: 137 mM NaCl, 5.4 mM KCl, 1.0 mM MgCl_2_, 0.33 mM NaH_2_PO_4_, 10 mM D-glucose, 10 mM HEPES, pH 7.35. Then, hearts were digested with collagenase (type II, 1.0 mg/mL, Worthington) Ca^2+^-free Tyrode’s solution for 10–12 minutes. Isolated cells were harvested in storage solution, which contained 120 mM potassium glutamate, 20 mM [EA5] KCl, 20 mM HEPES, 1.0 mM MgCl_2_, 10 mM D-glucose, 0.5 mM K-EGTA, and 0.1% bovine serum albumin. Na currents were recorded at room temperature by an Axopatch 200B amplifier (Molecular Devices) under whole-cell patch clamp mode. The bath solution contained 2.5 mM NaCl, 130 mM CsCl, 1 mM MgCl_2_, 1.2 mM CaCl_2_, 5 mM HEPES, 10 mM D-glucose, pH 7.35, with NaOH. 10 μM Verapamil (MilliporeSigma) and 40 μM Ni^2+^ were used externally to block L-type and T-type Ca^2+^ current. Pipette resistance was 1.2~2 MΩ after pipettes were filled with solution, which contained 2.5 mM NaCl, 1 mM CaCl_2_, 135 mM CsCl, 1 mM MgCl2, 4 mM MgATP, 10 mM HEPES, 10 mM EGTA, pH 7.2 with CsOH. Cell capacitance and series resistance were electronically compensated by 85%. Cells were holding at –80 mV. Steady-state activation was performed by a set of 100 ms test pulses to different voltages ranging from –120 mV to +30 mV after a 500 ms condition pulse at –120 mV. Steady-state inactivation was carried by a 50 ms test pulse at –30 mV after a family of 500 ms conditioning pulses at different voltage was applied. The start- to-start durations of these recording were set at 4 seconds for full channel recovery. Na conductance at each voltage was calculated by dividing the current by the driving force for sodium ions, and it was normalized to the maximum conductance for steady-state activation and inactivation analyses, both of which were estimated using Boltzmann’s equation. Recordings were made on atrial cardiomyocytes (*n* = 7 cells/mouse) both before and after saline and ^PYR^apelin^13^ administration. The effect of ^PYR^apelin^13^ (10 nM) was evaluated at 15 minutes after it was applied.

### Atrial histology and quantification.

Atrial tissues were embedded in paraffin, sectioned (5 μm), stained with Sirius red and fast green counterstain, and quantified as described previously (54). Briefly, images were digitized using a Leica camera (Leica Biosystems), and collagen deposition was quantified by measuring the red pixel content of digitized photos relative to the total tissue area (red and green pixels) using Adobe Photoshop 7 software.

For sedentary (*n* = 4) and swim-trained (*n* = 6) CD1 mice (Charles River), a different protocol was used, consistent with that from our previous work (29). Briefly, hearts were perfused with PBS containing 1% KCl followed by 4% paraformaldehyde in 0.1 M PBS. Hearts were paraffin embedded and 5 μm sections were stained with Picrosirius red for collagen quantification. Images were collected with a Nikon A1R confocal laser scanning microscope and quantified with ImageJ software (NIH) (55). Specifically, the image of the whole atrium was acquired through a combination of XY stitching and *Z*-stack function for individual sections. Collagen expression (percentage of tissue fibrosis) was calculated as the proportion of positive area to total tissue area through threshold adjustment of each section.

### Statistics.

Data are expressed as mean ± SEM unless specified otherwise. *n* refers to numbers of patients, animals, or cells where specified. Differences between LA myocytes before and after administration of ^PYR^apelin^13^ were analyzed by Wilcoxon signed-rank test or 2-tailed Student’s paired *t* test, depending on the outcome of a D’Agostino & Pearson omnibus normality test. The Mann-Whitney *U* test was used to compare AF durations as the data were not normally distributed according to a D’Agostino & Pearson omnibus normality test. For comparison of arrhythmic events between different treatment groups, a 2 × 2 contingency table using χ^2^ test without Yates’s correction was used. Exact *P* values are reported in the text. Other comparisons were made by using repeated-measures 2-way ANOVA and Fisher’s post hoc test. A *P* value less than 0.05 was considered significant.

### Study approval.

The study was approved by the Stanford Institutional Review Board and St. Michael’s Hospital Research Ethics Board (University of Toronto, Toronto, Ontario, Canada), and all patients gave written informed consent. Exercise-trained mice were housed at the Division of Comparative Medicine Vivaria (University of Toronto, Toronto, Canada) in accordance with the standards of the Canadian Council of Animal Care (Ottawa, Ontario, Canada). Ethical approval for all experiments was granted by the University of Toronto and York University.

## Author contributions

YMK and RL share co–first authorship, with authorship order based on initial study conception by YMK. YMK, RL, and JL performed the experiments described. AS, SV, and PHB were involved in ascertaining human samples. RL, TQ, and PHB developed the animal models. Monolayer recordings were performed by MS and MP. Optical mapping experiments were performed collaboratively with RL, HZ, JO, and PHB. EW performed atrial histology. YMK, RL, PHB, and EAA conceived of the experiments and wrote the manuscript.

## Supplementary Material

Supplemental data

## Figures and Tables

**Table 1:**
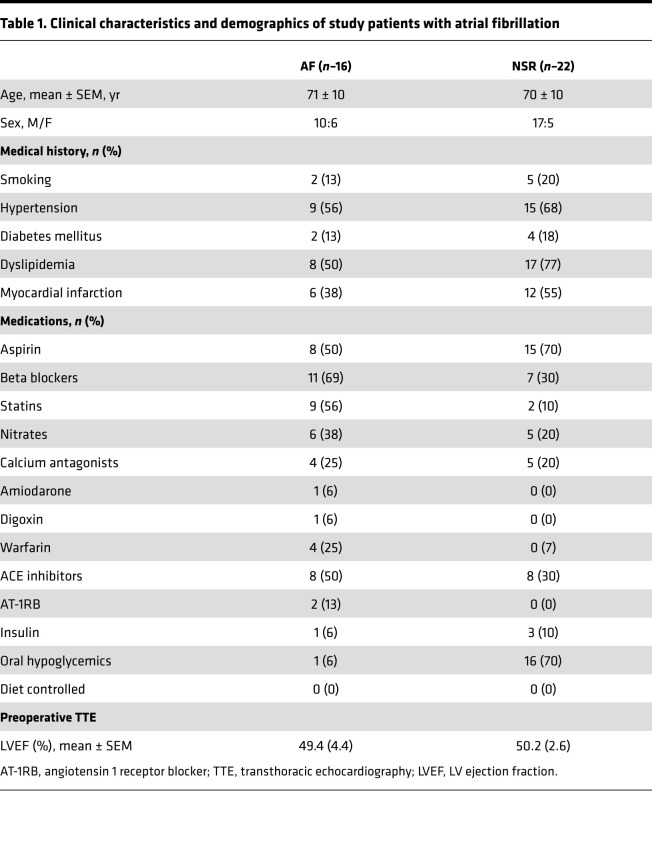
Clinical characteristics and demographics of study patients with atrial fibrillation

## Version 1 09/03/2020

Electronic publication
