# Supplementary material for: Apelin increases atrial conduction velocity, refractoriness, and prevents inducibility of atrial fibrillation
Source: JCI Insight. 2020 Sep 3;5(17):e126525. doi: 10.1172/jci.insight.126525 (PMC7526452; doi:10.1172/jci.insight.126525)
Supplement: Supplemental data [file jciinsight-5-126525-s083.pdf]

Supplementary Figures and Figure Legends

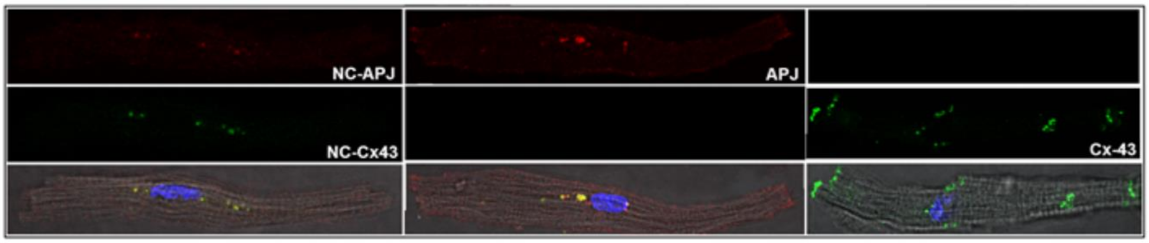

Supplementary Figure 1. Immunolocalization of the apelin receptor (APJ) in isolated human atrial myocytes. Immunolocalization of APJ and human connexin-43 (Cx-43) in freshly isolated human atrial myocytes. Immunocolocalization of human connexin-43 (Cx-43) at expected cellular domains of the myocyte demonstrate specificity of the immunofluorescence protocol. Negative control (NC) images were obtained by omission of the primary antibody. Phase contrast images illustrate nuclear counter-staining with DAPI (blue) and respective test epitopes. APJ in red fluorescence and Cx-43 in green fluorescence. Images were acquired using high power (100x) scanning laser confocal microscopy.

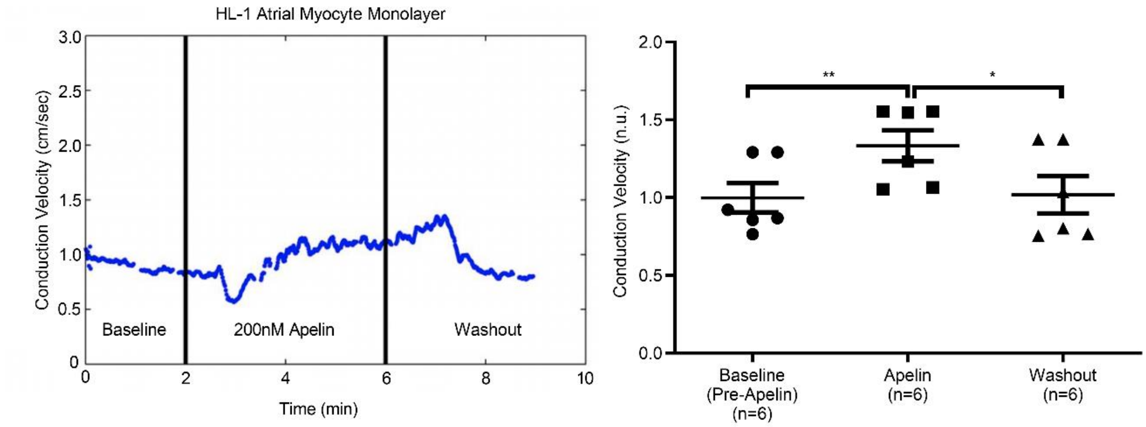

Supplementary Figure 2. Murine HL-1 atrial myocyte monolayer conduction velocity measurements. (A) Representative time course of changes in conduction velocity (CV)(cm/sec) in a cultured monolayer of murine HL-1 atrial myocytes showing an increase (P=0.006) in CV with apelin infusion (200nM), which is reversed upon saline washout. **(B)** Summary of changes in conduction velocity in murine HL-1 atrial myocyte monolayer, normalized to baseline (pre-apelin) (normalized units, n.u.), in response to variable apelin doses (200 and 1000 nM) and following washout. P values determined from repeated measures ANOVA comparing pre- and post- ^{PYR}Apelin^{13} administration and saline washout. *P<0.05, **P<0.01 as indicated.

Supplementary Methods

Murine HL-1 atrial myocyte monolayer conduction velocity measurements

The HL-1 cell line, derived from mouse atrial myocytes (10), was used in all described experiments. Prior to seeding the cells, the microelectrode arrays were sterilized with 70% ethanol and coated with an adhesion-promoting media containing 0.001% fibronectin (Sigma, St. Louis, MO, USA) and 0.02% gelatin (BD Biosciences, Sparks, MD, USA) and stored in a 37 °C incubator overnight. A suspension of HL-1 cells in culture media was obtained from a confluent flask of cells as described in (10). The culture medium consists of Claycomb media (JRH Biosciences, Lenexa, KS, USA), supplemented with 10% fetal bovine serum (JRH Biosciences), 100 µM norepinephrine (Sigma), 100 units/ml penicillin-streptomycin (Invitrogen Corp., Carlsbad, CA, USA), and 4 mM l-glutamine (Invitrogen). The gelatin/fibronectin solution was aspirated from the arrays and replaced with the cell suspension. The cells were plated at a density of approximately 1200 cells/mm^2. The cultures typically reached confluence and showed spontaneous electrical activity two days after plating. Electrical recordings were performed between days 2 and 10 after plating as described previously (10). Commercially available ^{PYR}Apelin^{13} was obtained from Bachem (H-4568, Torrance, CA) and a variable dose (200 or 1000 nM) was administered to test the exogenous effects of Apelin treatment on atrial conduction in this preparation.
